# Impermeable Urethral Stricture

**Published:** 1874-07

**Authors:** Oscar C. DeWolf

**Affiliations:** 441 West Washington St.; Chicago


ARTICLE II.—*Impermeable Urethral Stricture.* By OSCAR C. DE WOLF, M.D., of Chicago.

Prof. Syme announced his belief, twenty-five years ago, that all urethral strictures were permeable to the passage of a suitable and well directed sound, yet he felt obliged to make the confession that he occasionally met cases in which he was unable to pass an instrument of the smallest size from the external meatus to the bladder, *by means of manipulation only.* "On several occasions I have found it necessary to open the urethra anterior to the stricture so as to obtain the assistance of a finger placed in the canal to guide the point of the instrument."

The proposition is *not* that every urethral stricture can be passed by suitable instruments, for whenever the point of the sound is engaged in the constriction, and cannot be advanced without danger of piercing the canal, an incision may be made (external urethrotomy) so as to obtain the assistance of the eye and the finger placed in the canal, to guide the point of the instrument—and if one incision is made, it may be repeated whenever and wherever the point of the instrument is obstructed—but the question practically and honestly stated is this, are all urethral strictures permeable by a well selected instrument passed by manipulation from the external meatus towards the bladder? To this proposition there is but one answer. No.

What to do—and how to do it—with these cases, in which urinal retention may sometimes demand immediate relief, are questions which may embarrass the most skillful surgeon, and always alarm the younger and inexperienced practitioner.

I think they may be practically considered as of two classes, in both of which the urethra is absolutely impermeable to instruments of every description.

1. Those cases, chronic in their nature, in which there has been for a considerable time (perhaps for years) a persistently increasing and progressive thickening of the urethral walls, until the urine can only be extruded guttalim, or in a small dribble, the bladder thickened and contracted by the constant and violent action of its muscular coat, and the normal tissues of the perineum greatly indurated and perhaps traversed by sinuses and fistulæ. The following case is a fair type of this class, and also illustrates a physiological fact worthy of record.

Mr. P., æt. 55 years, farmer, of good general health, was injured stepping from his wagon by striking the perineum against the sharp edge of the iron rim of the wheel.

The urethra was not lacerated, and though there was slight retention for a few days he was very soon able to pass his water and resume his work. The urethra slowly but progressively narrowed, and after a time unavailing attempts were made to pass an instrument. In September, 1870, eight years subsequent to injury, he asked my attention. His condition was indeed pitiable. There had never been complete retention of urine save for a few days immediately following the injury, and yet he was now employed one-quarter of his time, night and day, in passing his water. He was emaciated and worn out. The walls of the bladder were thickened and contracted, and the perineum bulging and hard as cartilage. While attending to his general condition I gained time to attempt the introduction of an instrument.

After two weeks trial, in which nothing could possibly be passed beyond the peno-scrotal angle, I decided to open a passage with a bistoury to the bladder, and I can truly say that it was a difficult and trying operation.

Assisted by my friends, Drs. Fisk, Knowlton and Bonny, the patient was suitably placed, secured, and brought under the anæsthetic influence of sulph. æther. A silver catheter of full size was carried down to the obstruction and its point exposed by an external incision; the point was now turned out of the wound, and the short curved beak of the instrument used as a hook to steady the parts. Prolonged and fruitless effort was made to follow the tortuous and sinuous urethra, point by point, with a probe in extending the incision, but the induration and depth of the part, the very irregular and minute canal, and the difficulty of controlling the movement of the patient, rendered this impossible; finally, by an incision in the mesial line passing directly through the bulb, and with no attempt to find or follow the urethral canal, the indurated mass was divided back to the membranous urethra and the No. 12 catheter passed into the bladder. The wound (fully four inches deep) was packed with lint and the catheter tied in. With large doses of sulph. quinine and an occasional anodyne, the case progressed without a single annoyance. The bladder was washed twice a day with tepid water and the catheter retained in position ten days. It was readily introduced every day subsequent, by keeping the point well up against the upper surface of the new urethra.

In thirty days Mr. P. was about his house, the wound had entirely closed, and he was cautioned to pass a full sized sound once a week for a year. In a letter dated October 13th, 1873, three years after the operation, he reports: "My general health is good, the bladder retains the water as well as before my injury, and the water passes in a full stream. I have used no instrument for the last eighteen months."

It may be assumed, therefore, that whenever it is found impossible to "accurately and unswervingly" follow the track of the strictured urethra, the catheter may be placed in a new track and a new urethra will form about it, which in every way functionally performs the same duty as the normal one.

Mr. Wheelhouse, of Leeds (*British Medical Journal*, Feb. 10, 1870), suggests, that after the urethra is opened, just anterior to the stricture, and the short and sharply curved beak of the catheter, or sound, turned out of the cut so that the instrument may be used to draw forward, fix, and steady the urethra, that each lip of the opening, including the mucous membrane, should be seized with artery forceps, or a piece of thread passed through each side, with which to draw open and expose the canal.

I have found this suggestion of much service in attempting to follow the urethra with a fine probe. While the operation, under some circumstances, may be regarded as amongst the most difficult in the whole range of surgery, there seems to be no substitute to offer, unless the patient is to be allowed to continue his miserable existence to the end without aid, for though the bladder be reached, and its flow temporarily diverted, through the rectum or over the pubes (and in many cases this is impossible from its contracted and thickened condition), the urethral condition will not amend, but remains as stubbornly occluded as before.

The danger of hemorrhage attending or following the operation need not alarm, though it should be carefully watched for and immediately checked if it occur. If the mesial line of the perineum be accurately followed in making the incision, that "bete noir" of urethral surgery—the bulb—may be forgotten or ignored. It will be remembered that the operation here referred to differs from that known as "Syme's operation for perineal section," in this, that Mr. Syme directed that a sound of some kind should be carried through the stricture into the bladder, and upon which the surgeon was to cut from without, while in the cases considered, no instrument can be passed. It is sufficient to say of Syme's operation, that the best recent authorities regard it with disfavor, as unnecessary, for whenever an instrument can be introduced through a stricture it can be treated by other and safer means.

2. In the second class of cases the retention of urine is generally complete, and the services of the surgeon are requested to relieve a distended bladder. The urethral narrowing has not, to a certain time, been sufficient to occlude the canal, but from a variety of causes, as the violent distention of the urethra behind the stricture by the efforts of the bladder to expel its contents, cold, a debauch, instrumental attempts to enter the bladder, etc., a hyperæmic and swollen condition of the urethral tissue at, or just posterior to, the stricture has been produced, and which acts as the immediate cause of retention. A recognition of this superimposed inflammatory state is important as a guide to treatment, which should comprise the two-fold object, the immediate one of relieving the bladder, and the more remote, the removal of the cause of retention by restoring the urethra to its normal condition.

Though the canal has become absolutely impermeable to instruments, yet if we are able to divert the flow of urine to another channel for a few days, and thus give the irritable and diseased urethra physiological rest—the natural means of restoration—we may in all probability succeed in passing a sound, and be able to treat the case successfully as one of simple stricture. Puncture of the bladder through the rectum, or over the pubes, is very especially applicable in these cases.

In the "Transactions of the Medico-Chirurgical Society of London, 1868," there is a paper by Mr. Cock, Senior Surgeon to Guy's Hospital, detailing his experience in forty cases of puncture of the bladder through the rectum, in which he says, "this operation is safe, easy of accomplishment, and without danger as to its consequences, and in cases of retention which resist ordinary treatment it is greatly to be preferred to long continued attempts at catheterism."

While I have never found great difficulty in securing the canula in the bladder—and even if it escape, the orifice is immediately closed by the muscular coat and the operation may be repeated—nor have I ever seen in my own practice any other than good result from this operation—yet I prefer the hypogastric puncture, and particularly so since the capillary aspirator has been suggested, and so successfully used for this purpose. Mr. Watelet assures us that the operation may be done three or four times a day, and is "absolutely devoid of danger."

Dr. James I. Little also records (*New York Medical Journal*, Nov., 1872,) a case of retention of urine, in which he punctured the bladder fourteen times above the pubes, until a catheter could be introduced by the urethra; "no tenderness followed the punctures, and in a few days all trace of them had disappeared." Dr. L. suggests that the punctures should be made on or near the median line, about one inch above the pubes, and when necessary to repeat should be made each time in a different place, about a line apart.

441 West Washington St.

